# Anonymous Real-Time Analytics Monitoring Solution for Decision Making Supported by Sentiment Analysis

**DOI:** 10.3390/s20164557

**Published:** 2020-08-14

**Authors:** Gildásio Antonio de Oliveira Júnior, Robson de Oliveira Albuquerque, César Augusto Borges de Andrade, Rafael Timóteo de Sousa, Ana Lucila Sandoval Orozco, Luis Javier García Villalba

**Affiliations:** 1 Cyber Security INCT Unit 6, Laboratory for Decision-Making Technologies (LATITUDE), Department of Electrical Engineering (ENE), Faculty of Technology, University of Brasília (UnB), 70910-900 Brasília-DF, Brazil; jrgildasio@gmail.com (G.A.d.O.J.); robson@redes.unb.br (R.d.O.A.); caborges72@gmail.com (C.A.B.d.A.); desousa@unb.br (R.T.d.S.J.); asandoval@redes.unb.br (A.L.S.O.); 2 Group of Analysis, Security and Systems (GASS), Department of Software Engineering and Artificial Intelligence (DISIA), Faculty of Computer Science and Engineering, Office 431, Universidad Complutense de Madrid (UCM), Calle Profesor José García Santesmases 9, Ciudad Universitaria, 28040 Madrid, Spain

**Keywords:** big data, botnet, monitoring, real-time visualization, sentiment analysis, text mining, social media, Twitter

## Abstract

Currently, social networks present information of great relevance to various government agencies and different types of companies, which need knowledge insights for their business strategies. From this point of view, an important technique for data analysis is to create and maintain an environment for collecting data and transforming them into intelligence information to enable analysts to observe the evolution of a given topic, elaborate the analysis hypothesis, identify botnets, and generate data to aid in the decision-making process. Focusing on collecting, analyzing, and supporting decision-making, this paper proposes an architecture designed to monitor and perform anonymous real-time searches in tweets to generate information allowing sentiment analysis on a given subject. Therefore, a technological structure and its implementation are defined, followed by processes for data collection and analysis. The results obtained indicate that the proposed solution provides a high capacity to collect, process, search, analyze, and view a large number of tweets in several languages, in real-time, with sentiment analysis capabilities, at a low cost of implementation and operation.

## 1. Introduction

The widespread use of social networks offers new information source opportunities for government agencies and for companies. Publishing or spreading an idea has become a common practice on social networks. The dissemination of individual opinion and expression through the various channels leads to the formation of bases that are useful for generating knowledge about the events and changes of the current world. Monitoring, analyzing data and sentiments about companies, predicting scenarios that impact public opinion on strikes, protests, marketing, cyber-attacks, elections, military operations, and market research are examples of how information extracted from social networks can anticipate possible scenarios within a given context of interest. This kind of ability allows the concerned parties to understand a particular subject under discussion and to raise the possibilities of a particular issue.

With the creation of Web 2.0, social media presented a new way of obtaining data and developing applications. People began to share their experiences and opinions in large quantities online [1]. According to Pereira-Kohatsu et al. [2], social media also represents sensors in the real world that can be used to measure the pulse of society. This huge mass of data, available to system developers through Application Programming Interfaces (API) (an application programming interface is a set of routines and programming standards for software access), has been of great interest to researchers and attracts numerous studies on data mining, automated sentiment analysis, and visualization, among other research areas.

On Twitter (a microblogging platform launched in 2006 with over 25 million unique monthly visitors [3]), any user can post a short message (tweet) with a maximum length of 280 characters. There is a public timeline that broadcasts the tweets of all users around the world as an extensive real-time information stream of over one million messages per hour, especially during events that become meaningful due to social, economic, or political contexts. Russel [4] cited human curiosity, the need to share ideas and experiences, to ask questions, and to interact quickly as striking features of this social network. Twitter dynamically enables all these aspects to be accomplished with impressive speed. In addition, this social network has a difference compared with others because of its asymmetrical model for followers, as any user can keep up to date with the latest happenings even if they do not follow the author’s post, while on other social networks, like Facebook and LinkedIn, connection acceptance is required among users.

Due to the speed at which information is shared on the Internet, knowledge extraction techniques are used to automate search and processing of texts and, along with sentiment analysis techniques, make it possible to discover users’ judgment regarding products, services, and companies. Consequently, organizations are able to make improvements and adopt practices in line with the opinion of their target audience. Online platforms such as Twitter, which generate large amounts of data all the time—constituting a Big Data producer—have the potential to facilitate research over social phenomena based on sentiment analysis [5], as well as the search for new solutions to help extract useful knowledge from those large datasets.

Aiming to provide this capacity, this work proposes OctopusViz, a framework comprising a set of applications to monitor and collect a large number of tweets in real time, anonymously and online, and to automatically process, search, view, and sort message sentiments into three distinct categories: positive, negative, and neutral. OctopusViz proceeds by capturing the tweets according to the analyst’s interest and then classifies the sentiment of the tweets by using an algorithm that implements a lexical classification approach. Finally, the proposed framework displays the results in a graphical form, comparing different queries. The proposed framework enables real-time analyses and comparisons regarding metrics and sentiments of messages from various Twitter users, about certain topics, thus assisting in the process of smart decision-making in several environments and scenarios (commercial, police, military, etc.). It also allows easy identification of anomalies, and specifically outliers, which may be social bots trying to influence a particular subject. The main contributions of this work are detailed in Section 3.3.

This paper is organized as follows: Section 2 presents some basic concepts that are important for understanding the purpose of this work; Section 3 presents reviews of the state-of-the-art and some related work, as well as the main contributions of the proposed framework; Section 4 details the proposed environment architecture and the framework model; Section 5 gives a description of the implementation stages; Section 6 describes in detail a case study in which data were collected by the framework and further analyzed; Section 7 presents some work on the implications of adversary attacks on sentiment analysis; and finally, Section 8 presents the final considerations and proposals for future work.

## 2. Basic Concepts

This section introduces the concepts of data visualization and analysis, social bots, and anonymity systems that are used by the OctopusViz framework to assist in data presentation, understanding, and collection.

### 2.1. Data Visualization

Data visualization can be defined as a graphical representation of information and data [6]. It transforms data, information, and knowledge into a form in which the human visual system perceives embedded information [7]. According to Ward [6], visualization is important because “we are visual beings who use sight as one of our main senses for understanding information”. Therefore, the purpose of visualization is to aid data understanding by leveraging the human visual system’s ability to recognize patterns, perceive trends, and identify outliers [8]. If visualizations are well drafted, they can improve data understanding and give people an immediate and deep impression. Instead of subjecting people to the complex story reading process, we can get straight to the point [9].

Visualization has advanced with new ways of collection, manipulation, and interaction of data, blending with other fields and processes. In this context, visualization has also been a tool for data scientists. According to Gray et al. [9], at the reporting stage, visualizations may help journalists identify unusual topics, issues, trends, and deviations, find typical examples, and even suggest gaps and omissions in the reports. In addition, visualizations also perform various roles in the publication, as they more convincingly illustrate a point in the story, remove unnecessary technical information, and suggest transparency to readers about the reporting process.

### 2.2. Social Media Data Analysis

Data professionals have a plethora of computational tools available to assist them with the collection, cleaning, analysis, and presentation of data. Examples of those tools, such as Google Sheets, Web Scraper, OpenRefine, Infogram, Quadrigam, Google Analytics, Tableau, Gephi, etc., are abundant. However, according to Brooks [10], design incompatibilities and limitations may require specialized skills from these professionals, such as making extensive adaptations, finding fragile solutions, or changing contexts that might prevent their progress. This may restrict the participation of individuals in this emerging research area.

Some researchers have conducted studies to understand data analysis practices in various domains, such as intelligence analysis [11]; however, like other authors [10,12,13], we focus on the data analysis practices of scientists who work with social media data. Specifically in this study, we focus on data analysis practices of professionals who work with social media data in advising decision-makers.

Thus, social media data researchers face some methodological and technical barriers and questions about how social data online research should be conducted, ensuring, for example, validity, ethics, and reproducibility. Therefore, the design of data analysis tools is an interdisciplinary challenge that requires an understanding of the domain, as the data analyst works in other technical fields such as media and journalism [10,14].

### 2.3. Social Bots

According to Ferrara et al. [15], a social bot is a computer algorithm that automatically produces content and interacts with humans on social media, trying to mimic and possibly alter their behavior. Social bots have populated social media platforms in recent years.

For Kitzie [16], in addition to potentially endangering democracy, causing panic during emergencies, and affecting the stock market, social bots can undermine our society in even more subtle ways. A recent study proved the social media users’ vulnerability to a social botnet designed to expose private information, such as phone numbers and addresses [17].

According to Hwang et al. [18], that kind of vulnerability can be exploited by cybercriminals and erode trust in social media. Bots can also hinder the advancement of public policy by creating the impression of an opposing grassroots movement or contribute to the strong polarization of political discussion observed in social media [19]. They can alter the perception of the influence of social media, artificially increasing some people’s audiences [20], or they can ruin a company’s reputation for business or political purposes [21]. A recent study showed that emotions are contagious on social networks [22]: indescribable bots could easily infiltrate a population of unconscious zombies.

### 2.4. Anonymity Systems

According to Edman et al. [23], anonymity systems provide a “disassociation” between sender and recipients and between the receiver and senders. They fall into two classifications: (i) high-latency, used in message-based applications that tolerate delays; and (ii) low-latency for real-time applications [23].

Virtual Private Networks (VPNs) are considered low-latency anonymity systems. Using a VPN transfers user’s and Internet Service Provider’s (ISP) trust to the VPN provider as the first line of identification will be the VPN output to the Internet. That, in a way, provides privacy [24]. This aspect is important because it does not interfere with the collection and also ensures that the behavior change of users of social networks will not be necessary, bringing greater reality to the publication sentiment.

According to Çalışkan et al. [25], VPNs also provide secure communication to ensure data traffic confidentiality. Thus, malicious users will only observe encrypted data. Communication integrity is also provided to ensure that any kind of traffic adulteration is detected and discarded [25].

Another highlight is that some VPN service providers support high bandwidth, low latency, high throughput, multiple concurrent connections, and payouts where source identification is not required [26].

## 3. State-of-the-Art Review and Related Work

This section presents state-of-the-art reviews and related work needed to develop and understand the proposed environment. Section 3.1 provides an overview of using text mining to conduct sentiment analysis from a variety of sources and also describes the challenges surrounding sentiment analysis tasks using Twitter as the data source. Section 3.2 cites some tools that were used to extract information and present data through visualization. Finally, Section 3.3 compares the various works with the proposed environment, citing the differences applied in the context of the contributions of this work.

### 3.1. Sentiment Analysis in Text Classification

Interest in the area of sentiment analysis has grown rapidly and aims to explore the visuals or texts present on different social media platforms through machine learning techniques, subjectivity analysis, or polarity calculations. Sentiment is often subtly or complexly represented in a text. An online user can use a wide variety of techniques to express his/her emotions. In addition, mixing objective and subjective information about a particular topic can generate noise, undermining the classification.

According to Anjaria and Guddeti [3], those noises, which are commonly found in most datasets available, range from simple expressions to complete sentences (stop words, emojis, ironies, etc.), making it necessary to clean or modify them with specific techniques. Thus, the task of automatically recognizing sentiments in texts becomes more complex.

Mladenovic et al. [27] presented a model that uses various language resources: morphological dictionaries, sentiment lexicon, lexicon of markers, and a WordNet-based ontology or classification of statements into ironic and non-ironic. The authors performed the evaluation on two collections of tweets that had been manually annotated according to irony. These collections of tweets are in the Serbian language. The best results of the developed classifier (precision = 68.6%, acc = 86.1%) were achieved with a set of five features: antonymous pairs in which one member has a positive sentiment polarity (PPR), the polarity of positive sentiment words (PSP), Parts-Of-Speech tags of words (POS), ordered sequence of sentiment tags (OSA), and irony Markers (M).

In their work, Gomes et al. [28] applied text mining to extract knowledge from the news about the Portuguese economy. The authors proposed a model for sentiment analysis that polarizes the news into positive, negative, or neutral and provides a document with procedures for organizations to extract knowledge from textual data. Thus, they visited websites with information about the economy of their country to represent the sentiment expressed and to analyze the published texts.

Rodrigues Barbosa et al. [29] used text mining processes to explore tweets that spoke about the Brazilian presidential elections in 2010 to trace the online sentiment of the population expressed in tweets, classifying them into positive, negative, and neutral, and to correlate the ranking of tweets to the events occurring in Brazil at the time of the elections, such as political debates, for example. Rodrigues Barbosa et al. [29] pointed out that Twitter’s interaction model induces users to continually share and express their opinions and feelings, which are propagated to their followers. However, determining the sentiment each tweet expresses can be a laborious task, prone to errors and ambiguity. Getting around these challenges, the work in [29] explored hashtags. In this particular case, hashtags were used to determine the sentiment expressed by Twitter users in the tweets referring to the Brazilian presidential election in 2010. The hashtags’ classification into some sentiment indicating its polarity was done manually.

Despite the use of various machine learning techniques and sentiment analysis tools during elections, there is a need for a cutting edge approach. To deal with these challenges, Hasan et al. [30] tried to contribute to the field including the adoption of a hybrid approach, involving three different sentiment analyzers, SentiWordNet, Word Sense Disambiguation (WSD), and TextBlob, to calculate the polarity and subjectivity of tweets; and two machine learning classifiers, naive Bayes and Support Vector Machines (SVM). The results showed that TextBlob and WSD were better compared to SentiWordNet when used with the SVN classifier to predict electoral sentiments. The WSD had a higher rate of accuracy for predicting sentiment when the naive Bayes classifier was applied.

Kunal et al. [31] proposed using Python, Tweepy, and TextBlob libraries to access and rate tweets using the naive Bayes algorithm. This proposal is intended to facilitate the process of analyzing, summarizing, and classifying tweets. Although not providing visualization mechanisms, the project provides real-time sentiment analysis of any community, government, religion, celebrity, or politician around the world at any given moment. The authors used the Rapid Miner Tool to compare decision tree and naive Bayes with a static dataset “Titanic” available in Rapid Miner. Naive Bayes was found to have an accuracy of 92.58% and decision tree only 79.04%. With these comparative results, naive Bayes was the best choice for classification in the case presented.

In Cerón-Guzmán and León-Guzmán’s work [32], the authors collected a dataset related to the 2014 Colombian presidential election tweets, and a supervised learning technique was implemented in a labeled collection of users to distinguish spammer from non-spammer accounts. They developed and applied a sentiment analysis system to investigate the potential of social media for voting intent inference. According to the experimental results, inference methods based on Twitter data were not consistent, although the proposed inference method achieved lower mean absolute error and correctly ranked the candidates with the most votes in the first round of elections.

In Tumitan and Becker [33], the SVM algorithm combined with the Sentilex dictionary sorted sentiments from tweets for political trend analysis. The study used the dataset of the Brazilian presidential elections of 2010, and the accuracy reached was 81.37%.

In Praciano et al. [34], a framework for space-time trend analysis of the Brazilian presidential elections based on Twitter data was proposed. Experimental results showed that the proposed framework was very effective at predicting election results, as well as providing the geolocation timestamp and tweet, with an accuracy close to 90% when the Support Vector Machine (SVM) algorithm was applied for sentiment classification.

### 3.2. Visualization Review

In general, data visualization is a graphical representation of information and data. Using visual elements, such as diagrams, graphics, and maps, data visualization is an accessible way to see and understand exceptions, trends, and patterns, which otherwise would not be easily perceived. Big Data tools and data visualization technologies are essential for analyzing huge amounts of information in real time and making data-driven decisions.

Several search systems have been developed for Twitter data mining. Diakopoulos et al. [12] developed a tool (Vox Civitas) for mining current events that aimed to support journalists while extracting news from Twitter that aggregated data. The user interface was specifically designed to enable the journalistic investigation of real-time responses to news events. Similarly, Marcus et al. [35] stated that TwitInfo allows users to explore real-time events occurring on Twitter. Both systems use timelines to extract notable elements based on tweets volume peaks and word frequency-based heuristics. Those two systems contain potentially valuable elements for a business analyst. However, none of them consider the geographic origin of the messages, thus losing a substantial level of context that could be used to collect business intelligence.

Social media offers potential opportunities for companies to extract business intelligence. Sijtsma et al. [36] introduced Tweetviz, an interactive tool to help companies extract actionable information from a large set of noisy messages on Twitter. Tweetviz identifies the sentiment of the tweet of the business location, identifies other business locations that Twitter users visit, and estimates some simple demographics of Twitter users who frequent a business. A case study to assess the system capacity indicated that Tweetviz can provide an overview of the issues and businesses of a company, as well as information that helps users create customer profiles. The goal of this research [36] was to leverage geographic information to provide actionable location-specific information.

Oliveira Junior et al. [37] featured an environment called HoneySELK for searching for and viewing cyber attacks in real-time. HoneySELK uses the ELK stack to perform distributed storage of the complete structure of real-time attack monitoring data, with georeferencing data, statistics, and graphs, indicating diverse relationships that aid in the identification and attackers’ modus operandi.

Pimenta Rodrigues et al. [38] applied Deep Packet Inspection (DPI) techniques to detect anomalies and evaluate different attacks on network traffic destined for a High Interactivity Honeynet. Based on the collected data and through the ELK Stack, it was possible to generate statistics of users, services, passwords used, and IP address distribution.

### 3.3. Main Contribution of This Work

Given that Twitter presents social networking features interesting for mining, it is the chosen system for detecting users’ opinions. The tweets that indicate the sentiments of their authors, logical user statistics, hashtags, retweets, mentions, number of likes, and user mapping through graphs are extracted and analyzed according to the analyst’s interest. In this work, a text mining process similar to Gomes et al. [28] is used; however, the application is on the social network Twitter. The choice of using this social network is due to its global reach, which has millions of registered users. The texts to be mined make up a tweet, which is a string published by users and may contain other types of attached data.

This paper proposes functions analogous to those presented in [29,30,32,33,34] to determine the sentiment expressed by users, correlating the results to the facts that occurred in a certain period focused on a context of interest. However, it differs from the aforementioned works due to its operation being executed in real time, besides considering other words of a tweet that may express a sentiment, even if they are not marked with a hashtag.

Unlike the works by [12,31,35], the classification is done through an algorithm that uses a lexical approach, which classifies in real time the opinions of Twitter users, resulting in a categorization similar to the cited papers: positive, negative, and neutral (see Section 5.3).

As for the visualization aspect, this work has functions similar to the works of Sijtsma et al. [36], Oliveira Júnior et al. [37], and Pimenta Rodrigues et al. [38] for the set of viewing options. For example, Section 6.9 presents a quick and practical method using visualization tools for identifying social bots by analyzing outliers. Unlike all the works presented above, this paper prioritizes the anonymity of the analyst by using a VPN for data collection. Table 1 highlights the differences of OctopusViz regarding the cited related works.

## 4. Problem Statement and Proposed Solution

According to Gomes et al. [28], with the popularization of the Internet, people generate huge amounts of data every second. The challenge/problem is to know how to manipulate this large amount of information generated and to investigate how organizations can benefit from these data, considering that much of this knowledge is contained in texts, besides being able to perform the analysis of the data in real time.

**Figure 1:**
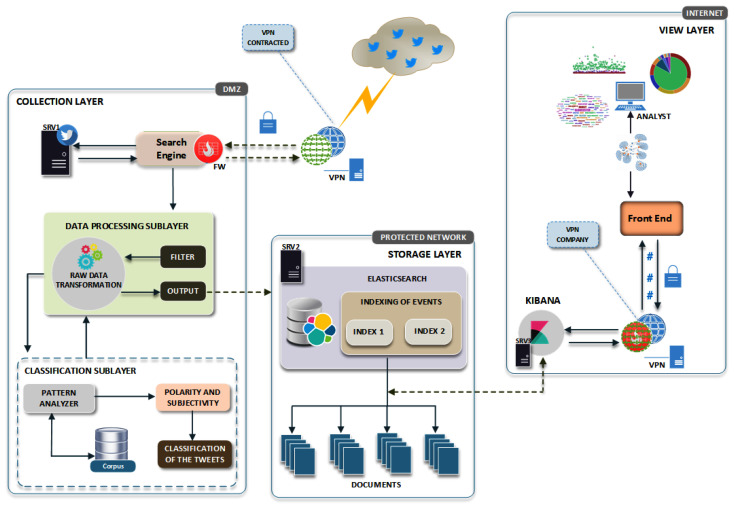
OctopusViz architecture.

This section describes aspects related to the development of the proposed environment named OctopusViz, whose proposed architecture (Figure 1) aims to collect, process, research, analyze, and visualize real-time Twitter topics, contexts, and trends, according to the need of the area interested in a particular subject.

### 4.1. Problem Definition

Due to the exponential growth of social media around the world, more and more government agencies and businesses are relying on information for their business strategies. Thus, it is essential to develop techniques to monitor and observe the evolution of a given theme, generating data that collaborate in the decision-making process.

In social media analysis, it is considered that an analyst should take into account and use all legal measures within his or her power to identify potential influencers’ and users’ sentiment about a particular subject. Therefore, this work proposes a solution that aims to contribute with logical statistics, sentiment analysis, the identification of tweets, retweets, hashtags, mentions, the amount of likes, user mapping through graphs, and construction of a real, up-to-date basis for research and analysis.

Such possibilities justify the present work, considering the importance of having an environment to monitor and analyze data and sentiments on various topics, such as strikes, elections, companies, marketing, protests, cyber-attacks, military operations, and market research. This type of capacity allows anticipating possible scenarios for advising on the decision-making process.

### 4.2. Proposed Environment Architecture

As a fundamental requirement, and for security and privacy reasons, the environment should ensure that the source of the collection is anonymous in order to not create an opportunity for data change trends or the possibility of influencing the data source. Therefore, one of the ways to achieve this requirement is by using a VPN, so that any collecting sources are not easily identified, avoiding the risk of data contamination by the source. Figure 1 shows the proposed environment’s architecture.

The environment’s physical architecture employs a host connected in a Demilitarized Zone (DMZ) and to a protected network of the University of Brasília (UnB) Research Laboratory. In this host, there are three virtual machines.

Hypervisor XenServer was configured to create logical and routing infrastructure for the environment of [39]. The entire project structure was made in just one host (Dell PowerEdge R730). The configuration and management of guest systems in XenServer is done through XenCenter. Table 2 presents the characteristics of the host and the hypervisor used for the development of the proposed architecture. Table 3 shows the guest systems’ configuration.

The environment is designed to have three distinct layers (Figure 1): (i) The collection layer captures tweets in real time according to keywords entered in the application. That layer also has (i.i) the processing sublayer, which transforms raw data into the information of interest according to the filters, and (i.ii) the classification sublayer, which performs sentiment analysis and uses computational resources to identify users’ public opinion. (ii) The distributed storage layer indexes and fetches tweets received from the capture layer. Finally, (iii) the visualization layer is responsible for data presentation to facilitate analysts’ interpretation.

The collection and visualization layers and the data processing and classification sublayers are configured on the lab DMZ network. The distributed storage layer is on the protected network, with restricted and controlled access.

To ensure anonymity, the collection layer authenticates to a contracted VPN server during the research of this project. All tweets collected by the search engine are sent to the distributed storage, which uses Elasticsearch to support and index large volumes of data, as the works [37,38,40]. Data presentation with metrics, statistics, and graphs, indicating different relationships, is done through Kibana [37,38,40,41].

## 5. Description of the Implementation Phases

The development of the proposed architecture took place in five phases, with Phase 1 dealing with the data collection layer; Phase 2 with the data processing sublayer; Phase 3 with the classification sublayer; Phase 4 with the distributed storage layer; and Phase 5 with the real-time tweets’ visualization aspects. The details of each phase are explained below.

### 5.1. Phase 1: Data Collection Layer

This layer is intended to collect data of interest on the Twitter platform in real time. Authentication and data collection are done with the Tweepylibrary [42] set up in Python [43]. This API authenticates the Python client user through an application (keys and tokens) built on Twitter. In the architecture, the search engine is configured to authenticate with the VPN contracted by the project to ensure privacy and confidentiality [24].

### 5.2. Phase 2: Data Processing Sublayer

The processing sublayer transforms raw data into the data of interest. As there is no writing standard to be used on social networks, it was necessary to execute some procedures to obtain better results in the refinement of information. This process is done through the TextBloband NLTK [44] libraries in a Python script (data transformation and centralization). The TextBlob API works with Natural Language Processing (NLP), sentiment analysis, classification (naive Bayes and decision tree algorithms), tokenization, translation, and spelling [45].

#### 5.2.1. Translation and Correction of Textual Data

Before any processing, the language of each tweet is detected, translated, and automatically corrected for the English language. This translation is done dynamically by the Google Translate API through the methods *get_languages()*, *detect_language()*, and *translate()* [46]. The correction is done by the *correct()* method of the TextBlob library [45]. This procedure allows the developed proposal to be used with more than 100 languages and thousands of language pairs. Table 4 presents a function with the translation and correction methods used in the environment.

#### 5.2.2. Stop Words and Special Characters

This technique is applied during the pre-processing activity. Its purpose is to remove words that have no value for analysis, generally corresponding to articles, prepositions, punctuations, conjunctions, and pronouns. The corpus stop words and the methods *stopwords.words()* and *string.punctuation* of the NLTK library are used for this function [44]. Moreover, we also removed the URLs from the tweets, bearing in mind that these URLs direct to information that does not present data with requirements for the sentiment analysis in our work. Table 5 shows the special characters, punctuations, and some stop words that are removed during pre-processing. Table 6 shows an example of the function used to remove stop words and special characters.

#### 5.2.3. Tokenization

The identification of tokens (words) is an important pre-processing step that divides texts into words, phrases, or symbols. In this work, the method *textblob.tokenizers.WordTokenizer()* from the TextBlob library was used to split tweets into individual words. The generated words help in the analysis and execution of other tasks of the Classification Sublayer. Table 7 shows an example of the function used for tokenization.

### 5.3. Phase 3: Classification Sublayer

This layer’s main objective is to carry out the sentiment analysis of tweets to identify behaviors that may come to measure public opinion. The TextBlob library is configured to process the textual data (classification of tweets).

#### 5.3.1. Sentiment Analysis

The module *textblob.sentiments()* contains two implementations of sentiment analysis algorithms: (i) *PatternAnalyzer* based on the Patterns library [47] and (ii) *NaiveBayesAnalyzer* (an NLTK classifier [44] trained in a corpus of movie reviews [48]. In this work, the Pattern Analyzeralgorithm and a lexical corpus were used. After the transformation, the data are sent to the sentiment analyzer. In this phase, the Pattern Analyzer algorithm consults the lexical corpus and classifies tweets through polarity, subjectivity, and intensity. The polarity score is assigned within the range (−1.0, 1.0), where: (0.01, 1 = positive), (−0.01, −1 = negative), and (0.0 = neutral). Subjectivity works with an interval of (0.0, 1.0), with 0.0 being very objective and 1.0 being very subjective [45]. The *_text.py* class is responsible for calculating sentiment. Table 8 shows the function used to classify tweets according to the values of polarity and subjectivity.

#### 5.3.2. Lexical Dataset

The file *en-sentiment.xml* contains the dataset that is used as a lexicon to assign scores (polarity, subjectivity, intensity, and confidence) and the Parts-Of-Speech (POS) tagger on each word within each sentence to determine grammatical class (nouns, verbs, adjectives, adverbs, etc.). Each word in the lexical dataset has a score. A lexicon of sentiments can be used to discern objective facts from subjective opinions in the text. The characteristics of the lexical dataset are as follows:

- Document XML that includes four entries: polarity, subjectivity, intensity, and confidence;
- Adjectives have polarity (negative or positive −1.0 to +1.0) and subjectivity (objective or subjective, +0.0 to +1.0);
- The score of each word is defined according to the meaning of the sentence, for example ridiculous (regrettable) = negative and ridiculous (humorous) = positive;
- Uses the Penn Treebank [49] tag set to determine the grammatical class (POS tagger) of the words: NN = noun, JJ = adjective, VB = verb, RB= adverb, CC = conjunction, IN = preposition, and UH = interjection.

### 5.4. Phase 4: Distributed Storage Layer

This layer indexes and searches for large volumes of data. This process is done by a separate guest on the internal network through the Elasticsearch tool. At this stage, Elasticsearch stores the full structure of tweets’ real-time monitoring data with distributed storage, which helps to understand and interpret behaviors collected from Twitter [37,38,40].

### 5.5. Phase 5: Visualization Layer

Viewing tweets and retweets in the environment is intended to make it easier for analysts to interpret them so it may be possible to anticipate information and to propose efficient measures from a data interpretation standpoint. In addition, real-time monitoring enables the observation of tweets, retweets, mentions, hashtags, entity relationships through graphs, the amount of likes, georeferences, and user sentiments on a topic.

This process is done by the Kibana tool [40,41]. This tool provides a rich interface to enable advanced analytical queries, visualization, and interaction with data stored in Elasticsearch indexes [40].

## 6. Case Study: 2018 FIFA World Brazilian National Soccer Team Theme

Currently, Twitter offers new opportunities to government agencies and companies to extract great relevance information about their strategies of interest because freely sharing ideas and opinions on a large scale in this social network has become a common activity. Because of this kind of behavior, this work can be used by analysts to collect, search, analyze, and view Twitter data in real time, regardless of their activity. In the case of this proposal, unlike other open platforms studied, there is the application of sentiment analysis techniques, the collection’s anonymization, and the isolation of layers, transforming the concept into a modular solution, adding or removing components as needed.

It is important to mention that the tool developed and the methods applied for the current analysis of the case study are not restricted to this particular subject. We decided to use the Brazilian National Soccer Team case because it would bring a neutral view regarding politics, enterprises, religion, or color discussions, while being able to state the capacity of what was developed.

### 6.1. Data Collection

The environment underwent several implementation tests before being put into production to minimize errors and false positives. The solution was installed in the research laboratory of the University of Brasília. Data collection took place between 15 June and 31 July 2018. This collection aimed to observe through metrics, statistics, and sentiment the repercussions and users’ public opinion on Twitter on the topic “Brazilian National Soccer Team” during the 2018 FIFA World Cup games. The environment followed the Boolean logic used by the Twitter search function. The keywords used for the collection were: “seleção brasileira” (Brazilian National Soccer Team) or “seleção do brasil” (Brazilian Soccer Team).

### 6.2. General Collection Summary Presentation

**Figure 2:**
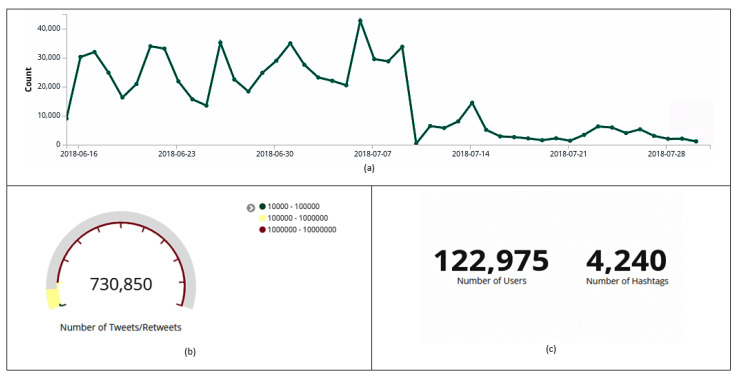
Data collected on Twitter between 15 June and 31 July 2018. Histogram with the amount of tweets and retweets collected per day (**a**). Total amount of tweets and retweets (**b**). Total number of users and hashtags (**c**).

Figure 2 represents through the visualization layer the total set of data collected by the environment. In Figure 2a, peaks of tweets and retweets that were posted on the Brazilian team match days (June 17th (31,957), June 22th (33,949), June 27th (35,204), July 2nd (34,968) and July 6th (25,506)) can be noticed, the most commented hashtag about the topic in the quarterfinals being detailed in Section 6.8, in Section 6.9, the identification of outliers (users with discrepant activities over a period of time), and in Section 6.10, the analysis of a botnet used to spread tweets. The biggest spike of published tweets and retweets was observed on July 7th (42,733), a day after the quarterfinals (July 6th), the match in which the Brazilian National Soccer Team was eliminated from the FIFA World Cup 2018. On the other hand, there is also a drop after July 10th, indicating that the theme “Brazilian National Soccer Team” was no longer so present on Twitter. It is also possible to identify that 122,975 users posted 730,850 posts and 4240 hashtags (Figure 2b,c).

### 6.3. Tweets’ and Retweets’ Analysis

Sorting messages into tweets and retweets is relevant so analysts can have an understanding of messages that can be studied according to interest. Furthermore, there is a consideration about the influence on a particular subject, i.e., a user can manipulate a particular theme by just sending a large amount of retweets.

**Figure 3:**
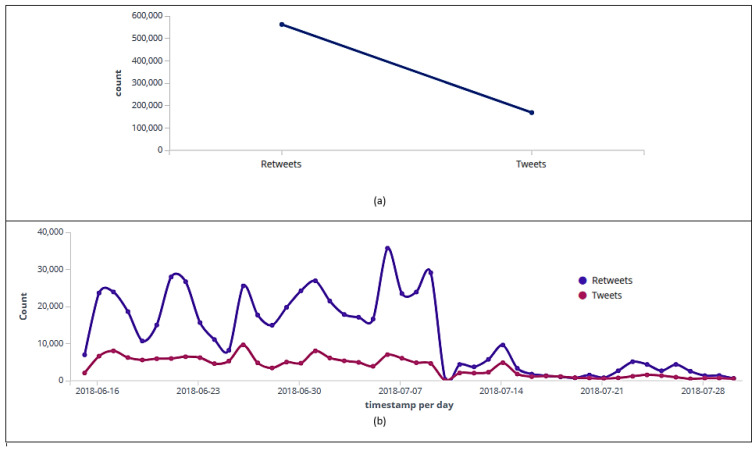
Data collected on Twitter between 15 June and 31 July 2018. Message classification into tweets and retweets (**a**). Histogram with the messages’ classification into tweets and retweets collected per day (**b**).

In Figure 3a, it can be noticed that out of the 730,850 posts, 168,509 were classified as tweets and 562,341 as retweets. Figure 3b displays the number of tweets and retweets published per day. During the entire collection period, every day, retweets outnumbered tweets, highlighting June 22nd (Round 2 of three in Group E: Brazil 2 × 0 Costa Rica) with 27,971 retweets, July 2nd (Round of 16: Brazil 2 × 0 Mexico) with 26,928 retweets, and July 7th (one day after quarterfinals: Brazil 1 × 2 Belgium) with 35,709 retweets. A difference (29,105 retweets-4647 tweets) also can be noticed on semifinal day (July 10th), when the team that eliminated Brazil in quarterfinals (Belgium) lost to France 1-0.

### 6.4. Hashtag Analysis

**Figure 4:**
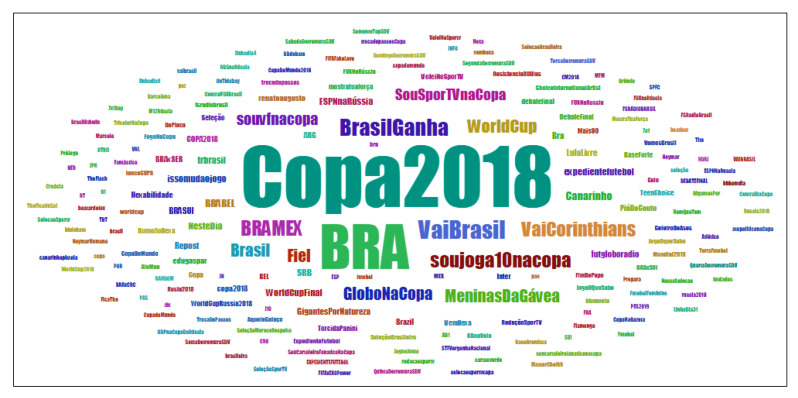
Cloud of words with the identification from the most referenced hashtags in tweets and retweets.

Information about hashtags is noted in the cloud of words in Figure 4 and detailed in Table 9, where *#Copa2018*, *#BRA*, *#VaiBrasil*, *#BrasilGanha*, and *#BRAMEX* stand out, those that were most referenced in tweets and retweets. It can also be noticed from Table 9 that those hashtags were more included in retweets than tweets.

It is worth noting that the special character *#* is removed from hashtags before indexing the data in the distributed storage layer (Section 5.4). This process is done in the data processing sublayer (Section 5.2), so tweets and retweets can be classified by the classification sublayer (Section 5.3).

#### Filter Application

**Figure 5:**
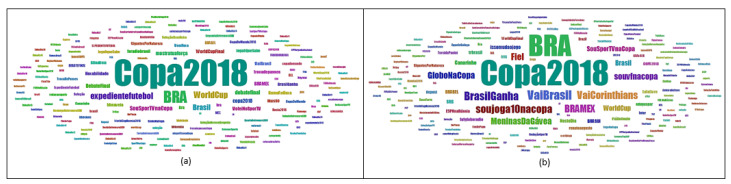
Cloud with the most referenced hashtags. (**a**) indicates tweets and (**b**) retweets.

Through the interaction provided by the tool, it was possible to apply filters to create and separate hashtags’ clouds by tweets and retweets. It was observed that the most referenced hashtags also depended on analyst interest in tweets (Figure 5a) or retweets (Figure 5b). It may be seen from the first two lines of the data represented in Table 10 that the hashtags *#Copa2018* and *#BRA* were the most commented on for both tweets and retweets. In the third line, it is observed that the hashtags change according to that interest.

### 6.5. User Analysis

Analyzing users is important from the perspective of an analyst, as there are many fake accounts on Twitter, some bots, and others having questionable purposes. In general, a regular user is unable to post many messages in a short period of time. Furthermore, it is common practice on Twitter to find fake accounts, bots, or strategies for spreading fake information (fake news) or even used to track users’ activities without their noticing.

**Figure 6:**
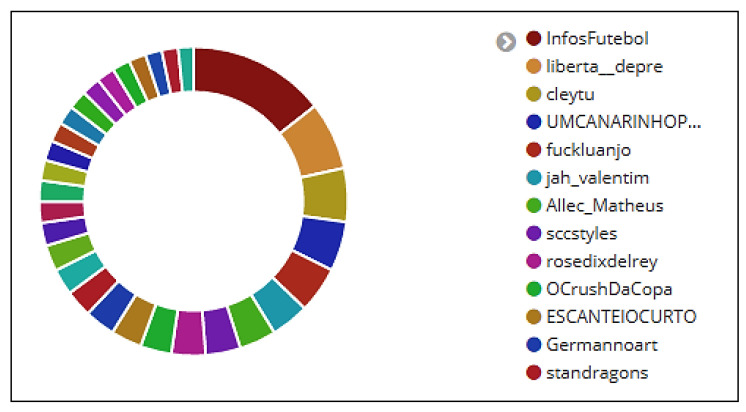
Users who posted the most tweets and retweets.

Figure 6 shows the thirteen users who sent the most tweets and retweets between 15 June and 31 July 2018. It is noted in Table 11 that during this period, the user who published the most retweets in the dataset was *InfosFuteboI*. Despite posting only 34 tweets, this account has amassed over 35,000 retweets (4857% of total data collected). It can also be seen from Table 11 that users *Allec_Matheus* and *rosedixdelrey* did not post tweets, but they retweeted a great deal.

**Figure 7:**
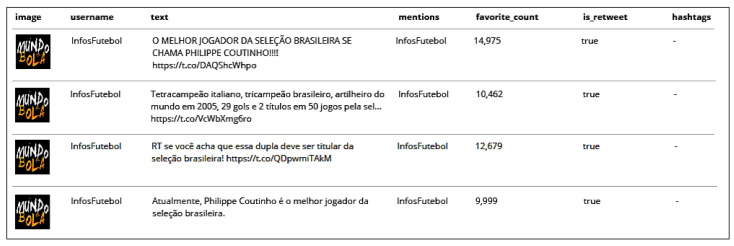
Retweets published by user InfosFutebol.

The environment also identifies mentions, likes, and hashtags quoted in each tweet and retweet. Four retweets that were posted by user *InfosFuteboI* can be observed in Figure 7. It is interesting to note how many likes (defined by the column *favorite_count*—14,975, 10,462, 12,679, and 9999) each retweet received.

### 6.6. Sentiment Analysis

Sentiment analysis is designed to rank users’ public opinion on tweets and retweets to identify the type of speech that allows specific decisions. This classification is made in the classification sublayer by the Pattern Analyzer algorithm (Section 5.3) and indexed on the distributed storage layer (Section 5.4).

**Figure 8:**
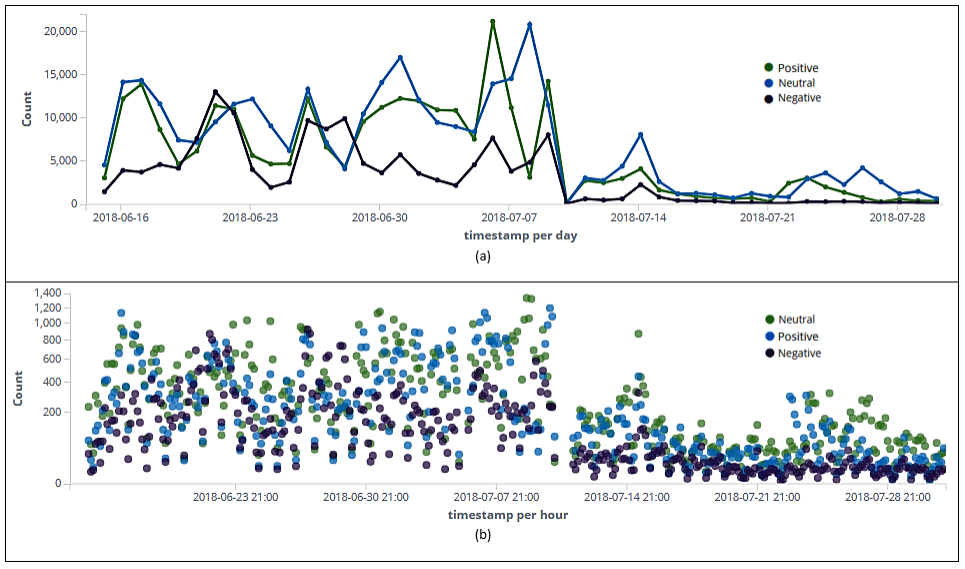
Classification (positive, negative, and neutral) of tweets and retweets per day (**a**). Classification (positive, negative, and neutral) of tweets and retweets per hour (**b**).

**Figure 9:**
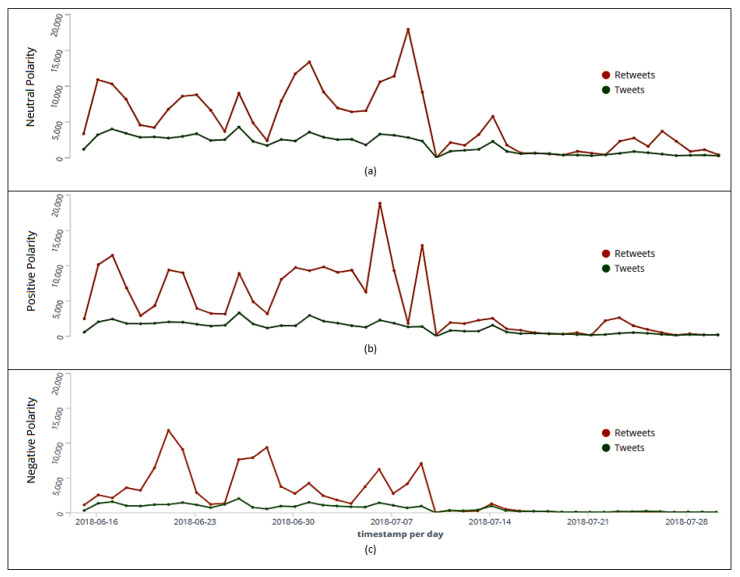
Ranking of tweets and retweets per day with neutral (**a**), positive (**b**), and negative (**c**) polarities.

**Figure 10:**
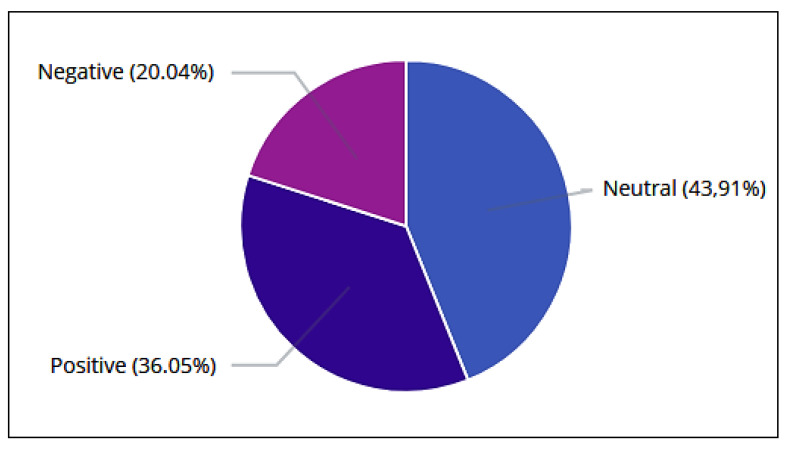
Overall ranking of tweets and retweets by the algorithm Pattern Analyzer.

Figure 8, Figure 9 and Figure 10 indicate user sentiment about the topic “Brazilian National Soccer Team” on tweets and retweets between 15 June and 31 July 2018. According to Figure 8a, the highest peak of positive-rated tweets and retweets (21,146) came on July 7th, the day after Belgium beat Brazil 2-1 in quarterfinals. It can be noted that even losing the match, the users were favorable to the Brazilian team. It is also noted in Figure 8a that the highest peak with negative polarity occurred on June 22nd (13,007) during Round 2 of three of Group E: Brazil 2 × 0 Costa Rica. Probably, this negative repercussion is due to the fact that Brazil tied the match on June 17th during Round 1 of three of Group E: Brazil 1 × 1 Switzerland.

In Figure 8b, it is also possible to spot peaks of tweets and retweets per hour during this time. It is observed that the highest peak with the positive polarity (1128) happened before the first match of the Brazilian National Soccer Team (6 p.m., June 16 th).

We also separated tweets from retweets to identify rating by polarity. Interestingly, throughout the collection period, the number of published retweets was higher across all polarities (neutral, Figure 9a, positive, Figure 9b, and negative, Figure 9c). Table 12 shows the days (June 22nd, July 7th, and July 9th) in which were posted the most tweets and retweets according to user sentiment analysis.

Considering the total number of tweets and retweets (730,850), sentiment analysis indicated through the algorithm Pattern Analyzer (Figure 10) that 36.05% (263,485) of users were in favor of the Brazilian selection, 20.04% (146,445) appeared to be against, and 43.91% (320,920) appeared to be neutral (Table 13).

### 6.7. Link Analysis

**Figure 11:**
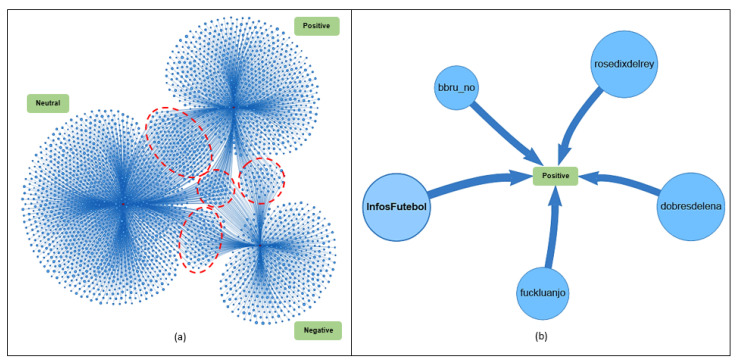
Relationship among the entities’ polarity (positive, negative, and neutral) and the user (**a**). Users who published the most tweets and retweets with positive polarity (**b**).

Link analysis aims to integrate information from various Twitter entities (users, hashtags, tweets, retweets, mentions, sentiment polarity, and images) to debug, organize, and interpret raw data, allowing the analyst to detect existing patterns and connections. Figure 11a presents a graph of the connection between the polarities (positive, negative, and neutral) and the user. For this analysis, we randomly selected 3000 indexed users in the environment. It can be seen from the graph in Figure 11a that more users were related to the neutral and positive polarities. It was also found that several users were related to two or three different polarities (positive, negative, and neutral). In this case, some tweets and retweets of those users were rated as positive and others as negative and neutral.

Figure 11b shows the positive polarity entity at the center of the graph. Under the Pattern Analyzer algorithm, users *InfosFutebol*, *fuckluanjo*, *rosedixdelrey*, *dobresdelena*, and *bbru_no* (closer to the center) posted more tweets and retweets in favor of the Brazilian team.

**Figure 12:**
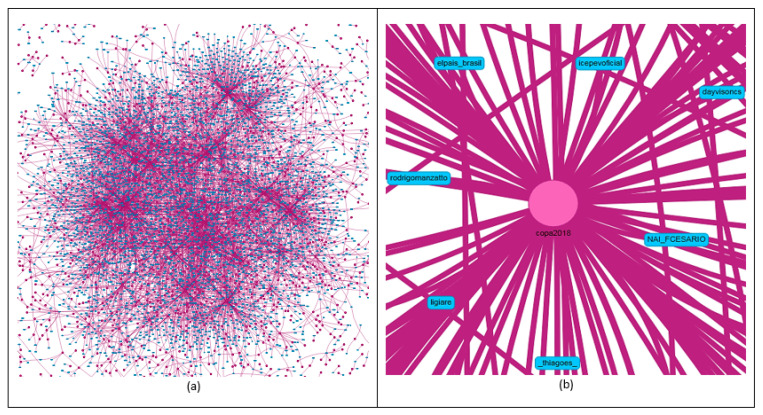
Relationship between hashtag and user entities (**a**). Hashtag most referenced in tweets and retweets (**b**).

Another link analysis was done between hashtag and user entities. In this analysis, all data indexed in the environment (from 15 June to 31 July 2018) were used. It is noted through Figure 12a the great difficulty in identifying those entities due to the number of existing relationships. The tool circumvents that problem by applying approximation filters. Thus, it was observed that the hashtag *#copa2018* appeared in the center of the graph as the most referenced in tweets and retweets (Figure 12b).

### 6.8. Analysis of the Most Commented on Hashtag in the Quarterfinals

**Figure 13:**
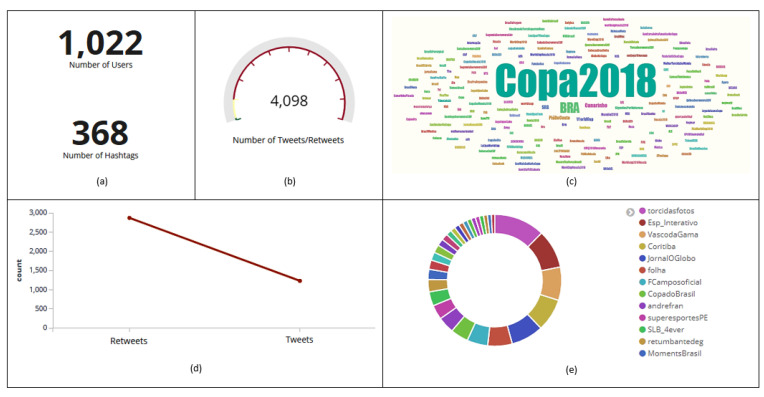
Number of users and hashtags (**a**), tweets and retweets (**b**). Most commented on hashtag (**c**). Rating of posts that have been included with that hashtag in tweets and retweets (**d**). Users who sent the most messages with that hashtag (**e**).

**Figure 14:**
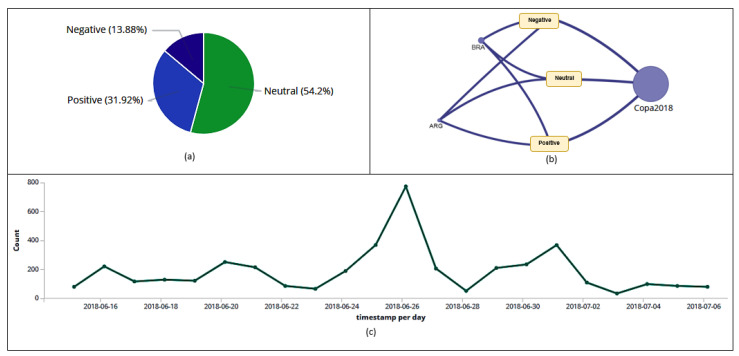
Polarity of tweets and retweets (**a**). Graph with the relationship between hashtag and polarity entities (**b**). Histogram with the amount of tweets and retweets collected per day with that hashtag (**c**).

On July 6th, during Brazil’s fifth match, in the quarterfinals, the hashtag *#Copa2018* stood out on the Dashboard. For more detailed information, a filter was applied to that hashtag. The filter result and its resulting information can be observed as follows: Figure 13a,b shows that 1022 users posted 4098 tweets and retweets with that hashtag; another 368 hashtags (Figure 13a) were related to *#Copa2018*; it can be noted in Figure 13d that 2869 posts with that hashtag were classified as retweets and 1229 as tweets; user *torcidasfotos* (Figure 13e) was the one who sent the most messages with that hashtag; it may be noticed in Figure 14a that 31.92% of users were in favor, 13.88% against, and 54.2% neutral; the hashtag *#Copa2018* (Figure 14b) was classified into three different polarities (positive, negative, and neutral); in Figure 14c, it may be spotted that the highest peak of tweets and retweets with that hashtag happened on June 27th (773).

### 6.9. Outliers Analysis

**Figure 15:**
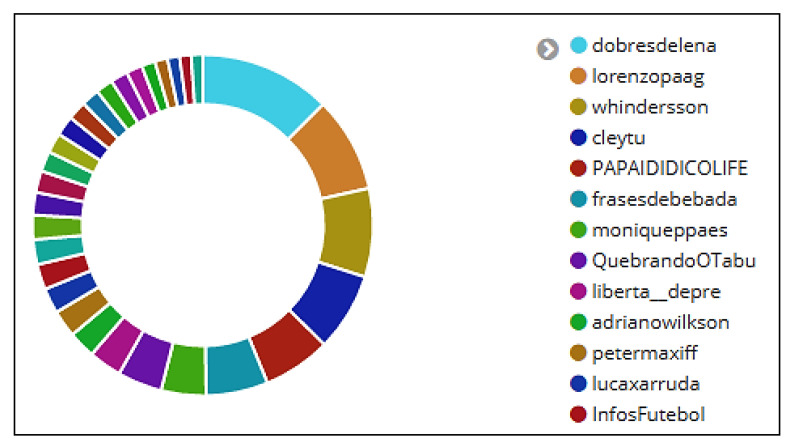
Data collected from Twitter between 15 June and 21 June 2018. Discrepant users.

For this work, we consider outliers elements those that do not follow a pattern of the set of users to which they were grouped according to the interest criteria analysis. They are users with discrepant activities for a period of time that require special attention as they usually produce values with unreliable effects. On June 21st, before the second match of the Brazilian National Soccer Team in the 2018 FIFA World Cup, it was found that the account *dobresdelena* presented a great distance from other users, being considered by the analysis an outlier (Figure 15). According to Table 14, users (*dobresdelena*, *lorenzopaag*, *whindersson*, *cleytu*, *adrianowilkson*, and *lacaxarruda*) did not post tweets. It was also noted that the user *dobresdelena* had posted 6723 retweets. A detailed analysis was conducted to try to identify the characteristics of that user.

**Figure 16:**
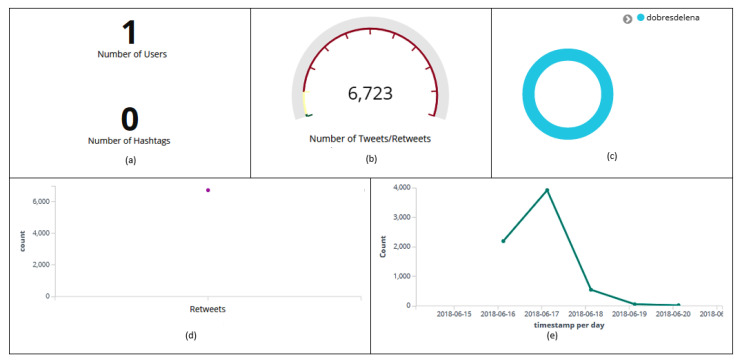
Dashboard with information about user *dobresdelena*. Number of hashtags (**a**), tweets and retweets (**b**). Discrepant user (**c**). Number of messages classified as retweets (**d**). Histogram with the amount of retweets collected per day (**e**).

**Figure 17:**
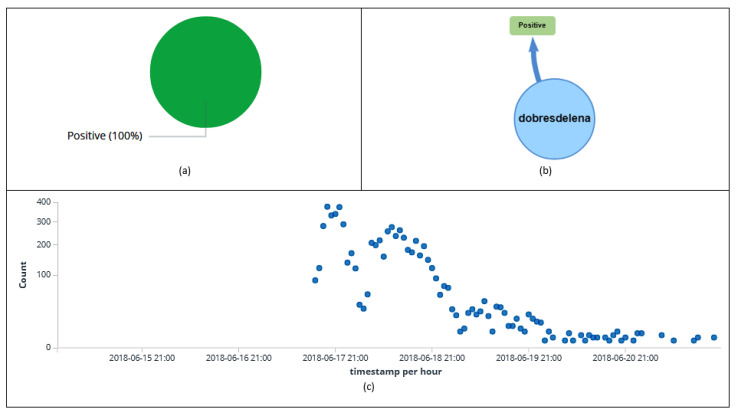
Information about *dobresdelena’s* retweet polarity (**a**). Relationship among the entities’ polarity (positive) and the user (**b**). Classification (positive) of retweets per hour (**c**).

Figure 16 and Figure 17 display information about the users, tweets, retweets, hashtags, and sentiment (message polarity).

Interestingly, all retweets were rated by the Patterns Analyzer algorithm as positive, influencing the analysis of user sentiment regarding “Brazilian National Soccer Team”. Information details may be observed as follows: in Figure 16a, it can be observed that user *dobresdelena* did not post any hashtag; Figure 16d shows that all posted messages were classified as retweets; it can be noticed that the highest peak of retweets (Figure 16e) happened on June 18th (3917); 100% of the retweets were classified as positives (Figure 17a,b); the highest peak of retweets (374) rated as positive happened on June 17th at 10 p.m. (Figure 17c).

### 6.10. Botnet Analysis

Botnets are algorithmically controlled accounts for performing repetitive functions (retweeting content, replying to and sending direct messages to new followers) or performing complex tasks (online conversations) on social media. The ability to remotely control large numbers of independent agents on Twitter has proven to be a powerful tool for performing activities, such as spam production, fake followers, debate manipulation, and public opinion [15].

**Figure 18:**
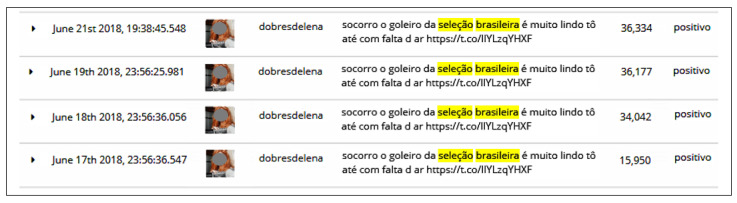
Retweets posted by the user *dobresdelena*.

In this scenario, we analyzed *dobresdelena’s* account on Twitter for relevant details to the interest analysis. Figure 18 shows that at the time of this analysis, the last retweet posted by that user had 36,334 likes. It can be noted that those retweets are always posted at the same time with a variation of seconds (June 17th, 23:56:36, June 18th, 23:56:36, and June 19th, 23:56:25). It can also be identified that in retweets, there is a link (https://t.co/lIYLzqYHXF) featuring three photos of the Brazilian national team goalkeeper, which were used as an account strategy to earn likes.

**Figure 19:**
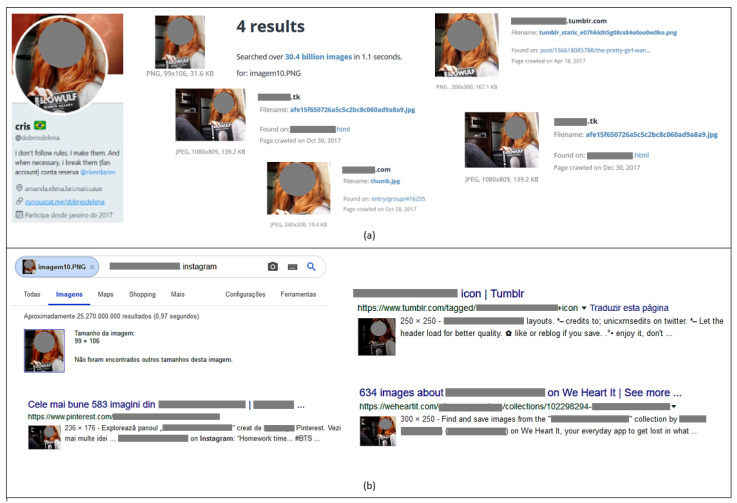
Reverse image analysis with the tools TinEye (**a**) and Google Images (**b**).

It is common for bots to use other people’s photos as avatars. In possession of that user’s image, it was subjected to analysis by Google Images [50] and TinEye [51] tools for the purpose of reverse searching and to find similar images in another place online. As shown in Figure 19a, the profile *dobresdelena* does not have a blue ticket to confirm the authenticity of its account on Twitter. Additionally, the tools TinEye and Google Images identified multiple results from sites that use that image with the same characteristics (Figure 19a,b).

**Figure 20:**
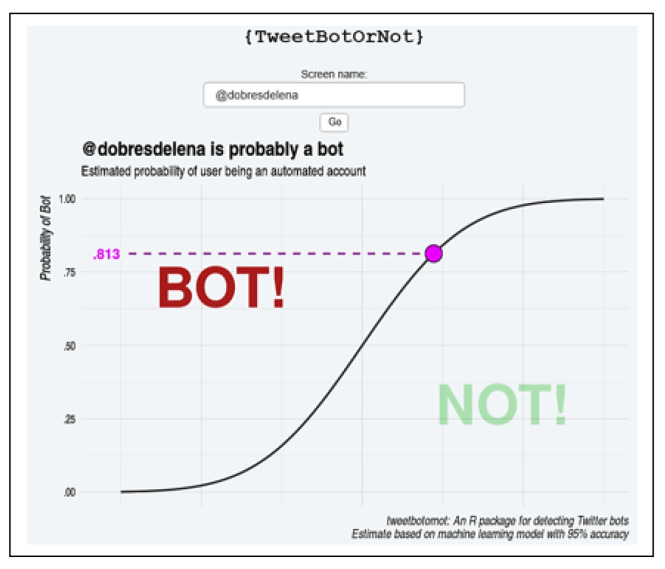
Analysis through the TweetBotOrNot tool.

The tool TweetBotOrNot [52] was also used to analyze the account *dobresdelena*. This tool uses machine learning to analyze metadata and classify account behavior on Twitter, indicating whether the user is a bot [52] or not. Figure 20 shows that TweetBotOrNot rated the account *dobresdelena* as a possible bot (0.813).

## 7. Implications of Attacks on Sentiment Analysis

Sentiment analysis techniques have been widely used in Natural Language Processing (NLP), among other fields. However, research on this topic has become a critical point with new methods of defense against adversarial attacks. In the natural language domain, small perturbations are clearly perceptible, and the replacement of a single word can drastically alter the result of the output of a sentiment classifier, changing the perception of analysts and influencing network users.

According to Hossein et al. [53], sentiment analysis techniques are vulnerable to the presence of intelligent and adaptive adversaries. The author proposed an attack based on adversary examples in a system that uses machine learning to automatically detect toxic language [54]. He showed that a small change in a highly toxic phrase can consistently reduce toxicity levels at the level of non-toxic phrases.

Tsai et al. [55] presented a method called “global search”, which consists of a white box attack algorithm. This method was compared with a simple misspelling noise and with another white box attack approach called “greedy search”. A sentiment classifier from the Convolutional Neural Network (CNN) was trained on the IMDB movie review dataset. Then, the attack methods were evaluated. As a result of the experiments, the proposed “global search” method generated more powerful adversarial examples with less deformation or less alteration in relation to the original text.

According to Li et al. [56], security vulnerabilities in Deep Learning-based Text Understanding (DLTU) are still largely unknown. The author showed that this technology is vulnerable to adversarial text attack. In their work, Li et al. presented TEXTBUGGER, an attack structure that generates adversarial texts. The efficiency of TEXTBUGGER was tested on a set of systems and services DLTU used for sentiment analysis and detection of toxic content. Results showed that TEXTBUGGER achieved 100% success on an IMDB dataset based on Amazon AWS in 4.61 s, preserving 97% of semantic similarity.

Samanta et al. [57], showed a new method for elaborating contradictory text samples, changing the original samples by deleting or replacing important words or adding new words to the text sample. Experimental results on the IMDB movie review dataset for sentiment analysis and in the Twitter dataset for gender detection showed the efficiency of the proposed method.

Alzantot et al. [58] proposed the generation of adversarial examples through the use of a population-based genetic algorithm to replace words with their synonyms, in order to generate semantically and syntactically similar adversary examples that deceive well-trained sentiment analysis in the first experiment and the textual linking models in the second experiment. A comparison between the success rate of the attack and the average percentage of modifications required by the attack genetics showed the efficiency of the method proposed in both experiments. A human validation showed that the generated examples were considered contradictory and perceptibly quite similar.

## 8. Conclusions

This paper proposes and describes a framework to analyze and follow in real time the evolution of topics of interest on Twitter.

The development of the proposed framework was divided into five phases, seeking the optimization of the aspects involved in the architecture. In the first phase, search engine settings were put in place to collect the data on Twitter, preserving the collector anonymity with the use of VPN. In the second phase, a Python script was implemented to perform the data transformation and centralization. In the third phase, an implementation of the Pattern Analyzer algorithm was used to perform sentiment analysis from tweets and to identify behaviors that can represent users’ public opinion. According to Sohangir et al. [59], the lexicon-based approach does not need training data, and so, it is favorable, particularly for tasks that involve high-dimensional data. In the fourth phase, the distributed storage of textual data was automated to aid the understanding and interpretation of the data collected from Twitter. In the fifth phase, tools were implemented to facilitate the analysts’ interpretation.

The solution tests showed that it was capable of capturing large amounts of tweets in real time. As a differential, the environment allows the performing of sentiment analysis, information extraction, user metrics and statistics, hashtags, tweets, retweets, social bots’ identification through outliers analysis, and quantitative data, which can be configured according to the needs and interests of those who need to analyze data at high volume and speed.

As a case study to validate the solution, Twitter data related to the “Brazilian National Soccer Team” were detailed. During the time of collection and analysis, it was possible to identify bots and the most commented hashtag in the fifth match of the Brazilian team in the 2018 FIFA World Cup quarterfinals. The analysis of the results indicated that such techniques allow using the proposed framework in several analysis applications. The Pattern Analyzer algorithm implemented in the *textblob.sentiments()* module of the TextBlob library proved to be effective, presenting in real time consistent results about the users’ sentiment (polarity and subjectivity of the text). The proposed solution also allows the viewing of tweets’ details for smart decisions without the risk of bots’ influence, as they can be effectively identified with the help of the tool.

### Future Work

As future work, we intend to create a new corpus in Portuguese, apply the framework to detect and classify users’ sentiment considering different types of irony, test other sentiment analyzers that use a lexical approach, use the framework dataset in machine learning algorithms to identify better results, to create sample methods and adversarial attack strategies to trick the classifier used, integrate data from other open sources, automate the bots’ identification process, and use the TOR network to collect and analyze data from the Deep Web and the Dark Web.

## Figures and Tables

**Table 1:** OctopusViz highlighted features compared to related works.

	Anonymization	Sentiment	Real Time	Distributed	Visualization
		Analysis	Operation	Storage	
OctopusViz	x	x	x	x	x
[12]	–	x	x	–	–
[29]	–	x	–	–	–
[30]	–	x	–	–	–
[31]	–	x	x	–	–
[32]	–	x	–	–	–
[33]	–	x	–	–	–
[34]	–	x	–	–	–
[35]	–	x	x	x	x
[36]	–	x	–	–	x
[37]	–	–	x	x	x
[38]	–	–	–	x	x

**Table 2:** Host features.

Server	Configuration
Dell PowerEdge R730	Intel Xeon processor E5-2690 v3 @ X5560 2.6 GHz, 48 cores with Intel VT technology, 128 GB RAM, 6 disks with 1TB configured with RAID 5, and 6 network cards 10/100/1000.
Hypervisor	XenServer 7.4, DBV 2018.0223.

**Table 3:** Guest systems and their settings.

Guest Systems	Configuration
Fw (Firewall)	2 core processor, 4 GB RAM, one 50 GB virtual disk, and 3 virtual network interfaces. pfSense-2.4.3-RELEASE version based on the FreeBSD Operating System.
Srv1 (Collection)	12 core processor, 16 GB RAM, one 50 GB virtual disk, and one virtual network interface. Operating system: Linux Debian Stretch 9.0 with the Python 3 programming language and libraries tweepy, json, time, elasticsearch, datatime, os, re, and textblob.
Srv2 (Storage)	12 core processor, 32 GB RAM, one 400 GB virtual disk, and one virtual network interface. Operating system: Linux Debian Stretch 9.0 with thee elasticsearch-6.2.4 service.
Srv3 (Visualization)	8 core processor, 8 GB RAM, one 50 GB virtual disk, and one virtual network interface. Operating system: Linux Debian Stretch 9.0 with kibana-6.2.4 service.

**Table 4:** Function for translation and correction of tweets.

**Example of Data Input in Portuguese**	O Brasil jogou muito bem contra a Costa Rica
	*tweet = TextBlob(“O Brasil jogou muito bem contra a Costa Rica")*
	*if tweet.detect_language() != ’en’:*
	*translate_to_english = TextBlob(str(tweet.translate(to=’en’)))*
**Data Preprocessing**	*correct_tweet = translate_to_english.correct()*
**(Translation and Correction)**	*print (correct_tweet)*
	*else:*
	*tweet.correct()*
	*print (tweet.correct())*
**Data Output**	*Brazil played very well against Costa Rich*

**Table 5:** Corpus words stop words and special characters.

Methods	Method Description	Data Output
*stopWords = set(stopwords.words(’english’))* *print(stopWords)*	Corpus words stop words	*[’i’, ’me’, ’my’, ’we’, ’our’, ’ours’, ’his’, ’y’, ’your’, ’it’]*
*string.punctuation*	Scores and special characters	’!"#$%&’()*+,-./:; <=>?@[]‘{|} ’

**Table 6:** Function for cleaning tweets.

**Example of Data Input**	Brazil is an excellent soccer team:) !!!
	*tweet = TextBlob(“Brazil is an excellent soccer team:) !!!")*
	*translation_correction(tweet)*
	*stopwords_english = stopwords.words(’english’)*
	*words = tweet.words*
**Data Preprocessing**	*words_clean = []*
**(Stop Words and Special Characters)**	*for word in words:*
	*if word not in stop words_english:*
	*if word not in string.punctuation:*
	*words_clean.append(word)*
	*print (words_clean)*
**Data Output**	*[’Brazil’, ’excellent’, ’soccer’, ’team’]*

**Table 7:** Function for tokenization of tweets.

**Example of Data Input**	Brazil played very well against Costa Rica
	*tweet = TextBlob(“Brazil played very well against Costa Rica")*
**Data Preprocessing**	*translation_correction(tweet)*
**(Tokenization)**	*tweet_clean_stop words(tweet)*
	*print (tweet.words)*
**Data Output**	*[’Brazil’, ’played’, ’very’, ’well’, ’against’, ’Costa’, ’Rica’]*

**Table 8:** Function for tweets’ classification.

**Example of Data Input**	Brazil is an excellent soccer team:) !!!
	*tweet = TextBlob(“Brazil is an excellent soccer team:) !!!")*
	*translation_correction(tweet)*
	*tweet_clean_stop words(tweet)*
	*tokenization(tweet)*
	*if tweet.sentiment.polarity > 0:*
	*print (tweet.sentiment)*
**Data Classification**	*print (’Polarity: Positive’)*
**(Polarity and Subjectivity)**	*elif tweet.sentiment.polarity == 0:*
	*print (tweet.sentiment)*
	*print (’Polarity: Neutral’)*
	*else:*
	*print (tweet.sentiment)*
	*print (’Polarity: Negative’)*
**Data Output**	*Sentiment(polarity = 0.98828125, subjectivity = 1.0)*
	*Polarity: Positive*

**Table 9:** The five most referenced hashtags between 15 June and 31 July 2018.

Hashtags	Tweets	Retweets	Total
#Copa2018	1356	3193	4549
#BRA	575	2701	3276
#VaiBrasil	100	1062	1162
#BrasilGanha	95	960	1055
#BRAMEX	123	821	944

**Table 10:** Hashtags most referenced by tweets or retweets.

Hashtags	Tweets	Hashtags	Retweets
#Copa2018	1356	#Copa2018	3193
#BRA	575	#BRA	2701
#WorldCup	277	#VaiBrasil	1062
#expedientefutebol	273	#BrasilGanha	960
#Brasil	238	#soujoga10nacopa	923

**Table 11:** Rating of tweets and retweets by user.

Users	Tweets	Retweets	Total
InfosFuteboI	34	35,504	35,538
liberta_depre	16	17,575	17,591
cleytu	2	13,856	13,858
UMCANARINHOPUTO	7	12,799	12,806
fuckluanjo	1	11,997	11,998
jah_valentim	2	9899	9901
Allec_Matheus	0	9529	9529
sccstyles	4	9046	9050
rosedixdelrey	0	8776	8776
OCrushDaCopa	1	8073	8074
ESCANTEIOCUTO	3	7986	7989
Germannoart	1	7901	7902
standragons	14	6971	6985

**Table 12:** Peak polarities of tweets and retweets per day.

Polarity	Day	Tweets	Retweets	Total
Neutral	July 9th	2824	17,946	20,770
Positive	July 7th	2291	18,855	21,146
Negative	June 22nd	1196	11,811	13,007

**Table 13:** Polarity of tweets and retweets.

Polarity	Tweets	Retweets	Total
Positive	53,993	209,492	263,485
Negative	31,230	115,215	146,445
Neutral	83,286	237,634	320,920

**Table 14:** Numbers of tweets and retweets by user.

Users	Tweets	Retweets	Total
dobresdelena	0	6723	6723
lorenzopaag	0	4877	4877
whindersson	0	4526	4526
cleytu	0	4003	4003
PAPAIDIDICOLIFE	2	3481	3483
frasesdebebada	1	3170	3171
moniqueppaes	1	2299	2300
QuebrandoOTabu	1	2279	2280
liberta_depre	5	1686	1691
adrianowilkson	0	1388	1388
petermaxiff	1	1385	1386
lacaxarruda	0	1296	1296
InfosFutebol	5	1287	1292

## Abbreviations

The following abbreviations are used in this manuscript:

API	Application Programming Interface
CNN	Convolutional Neural Network
DLTU	Deep Learning-based Text Understanding
DMZ	Demilitarized Zone
DPI	Deep Packet Inspection
ELK	Elasticsearch, Logstash, and Kibana
ISP	Internet Service Provider
NLP	Natural Language Processing
NLTK	Natural Language Toolkit
POS	Parts-Of-Speech
RSS	Rich Site Summary
SVM	Support Vector Machines
TOR	The Onion Router
URL	Uniform Resource Locator
VPN	Virtual Private Network
WSD	Word Sense Disambiguation
XML	Extensible Markup Language
